# Alcohol-induced autonomic dysfunction: a systematic review

**DOI:** 10.1007/s10286-019-00618-8

**Published:** 2019-06-20

**Authors:** Thomas Henry Julian, Rubiya Syeed, Nicholas Glascow, Panagiotis Zis

**Affiliations:** 1 The Medical School, University of Sheffield, Beech Hill Rd, Sheffield, S10 2RX UK; 2 Medical School, University of Cyprus, Nicosia, Cyprus; 3 Academic Department of Neurosciences, Sheffield Teaching Hospitals NHS Foundation Trust, Sheffield, UK

**Keywords:** Autonomic, Dysfunction, Alcohol, Alcoholic, Ethanol, Neurology

## Abstract

**Purpose:**

Autonomic dysfunction is a known consequence of chronic and excessive alcohol consumption. The aim of this systematic review was to characterise this phenomenon, describe the frequency at which it occurs and to explore the best management strategies.

**Methods:**

A systematic, computer-based search was conducted using the PubMed database. All studies identified by the search were evaluated independently by at least three authors. For inclusion, studies had to report human subjects consuming ethanol in excess. Case reports and non-original studies were excluded from this review.

**Results:**

A total of 55 studies were included in this review. According to cardiovascular reflex tests, 16–73% of chronic alcohol abusers suffer from autonomic dysfunction. The most commonly occurring symptom is erectile dysfunction, whilst other features such as postural dizziness are rare. The most important risk factor for this condition is total lifetime dose of ethanol, although there is mixed evidence supporting the role of other risk factors. The only management strategy currently explored in the literature is abstinence, which appears to lead to significant improvement in autonomic investigations.

**Conclusion:**

Current literature includes studies of highly heterogeneous populations, consuming differing volumes of alcohol over variable periods of time and utilising a number of different autonomic test batteries and criteria to diagnose autonomic dysfunction. Therefore, further research using homogeneous methods for measuring autonomic dysfunction in the field is needed. Despite this limitation, our review demonstrated that autonomic dysfunction is very common among alcohol abusers.

## Introduction

Chronic, excessive alcohol consumption is known to relate to diseases of both the central and peripheral nervous system. Such manifestations include dementia, delirium tremens, peripheral neuropathy and autonomic neuropathy [1, 2]. With respect to somatic and autonomic nervous damage, it is currently unclear whether the cause of dysfunction is the toxic impact of ethanol itself, or other confounding factors. However, for the purpose of this review the term “alcohol-related autonomic dysfunction” shall be used to indicate autonomic nervous system dysfunction related to chronic excessive alcohol use. The term “alcohol abuse” will be utilised henceforth to describe unhealthy, chronic, excessive patterns of alcohol use.

Whilst somatic neuropathy more commonly presents symptomatically, autonomic dysfunction is another important form of neurological compromise in the context of alcohol abuse and affects both the parasympathetic and sympathetic nervous system [3, 4]. Even in the absence of subjectively experienced phenomena, autonomic dysfunction is of clinical significance as it is associated with increased mortality [5]. At present, there is conflict in the literature in relation to the character of alcohol-related autonomic dysfunction and its features are yet to be properly dissected.

The aim of this review is to consider the evidence base describing the nature of alcohol-related autonomic dysfunction as well as its frequency, risk factors, prognosis and management.

## Methods

### Protocol registration

This review was prospectively registered to PROSPERO, an international prospective register of systematic reviews. The registration number for this review is CRD42018113087.

### Literature search strategy

A systematic search was performed on 10 June 2018 using the PubMed database. For the search, two medical subject heading (MeSH) terms were used. Term A was “alcohol OR alcoholic OR ethanol”. Term B was “neuropathy OR polyneuropathy OR dysautonomia OR autonomic dysfunction”. Human subject and English language filters were applied in our search. The reference lists of included articles were scanned for further articles which may fall within the scope of this review and were included where appropriate.

### Inclusion and exclusion criteria

Articles eligible to be included in this review were required to meet the following criteria:

1. The article discussed autonomic dysfunction related to chronic alcohol consumption. Studies with any reported measure of symptoms or objective findings of autonomic dysfunction were included. Studies were not restricted on the basis of their specific criteria for defining alcohol abuse.
2. The study was conducted using human subjects.
3. The article was written in English language.

Articles meeting the following criteria were excluded from our review:

1. Case reports.
2. Non-original articles (i.e. review articles, letters to the editor, expert opinion papers etc.).
3. Animal studies.
4. Duplicate articles (identical publications or referring to identical patient populations).
5. Studies of patient populations with other causes of autonomic dysfunction or with comorbidities which may cause autonomic dysfunction (e.g., diabetes, vasculitis, mechanical trauma).
6. Studies referring to large fibre peripheral neuropathy alone.
7. Articles which could not be obtained despite university interlibrary request, British Library request, and finally an attempt to contact the article authors.
8. Studies which contained subjects who had consumed any forms of alcohol other than ethanol (e.g. methanol) or who consumed illegally manufactured or homemade alcohol, as these beverages are likely to contain impurities and may also be more toxic.
9. Articles detailing pilot treatments that have not been replicated or further confirmed with larger studies.

All article abstracts were screened in triplicate in a blinded fashion using Rayyan software. Those found to meet any of the exclusion criteria were removed and any conflicts were settled by consensus during a face-to-face meeting in which the abstracts were re-read. All remaining papers were screened again as a full article by at least two authors and conflicts were settled as previously described.

### Data collection process

Data were extracted from each study in a structured coding scheme using Google Sheets and included population size, gender distribution, prevalence data, the nature of autonomic dysfunction, the means of diagnosis of autonomic dysfunction, risk factors, prognosis and response to treatment.

When there was uncertainty regarding how data should be interpreted or utilised, at least three authors discussed the study in question to ensure consensus.

### Synthesis of results

This study is reported in accordance with the preferred reporting items for systematic reviews and meta-analysis (PRISMA) guidelines [6]. The data is presented numerically where possible. The synthesis of the data is descriptive.

### Compliance with ethical guidelines

This article is based upon previously published studies. There are therefore no ethical concerns with regard to this study.

## Results

### Study characteristics

**Fig. 1:**
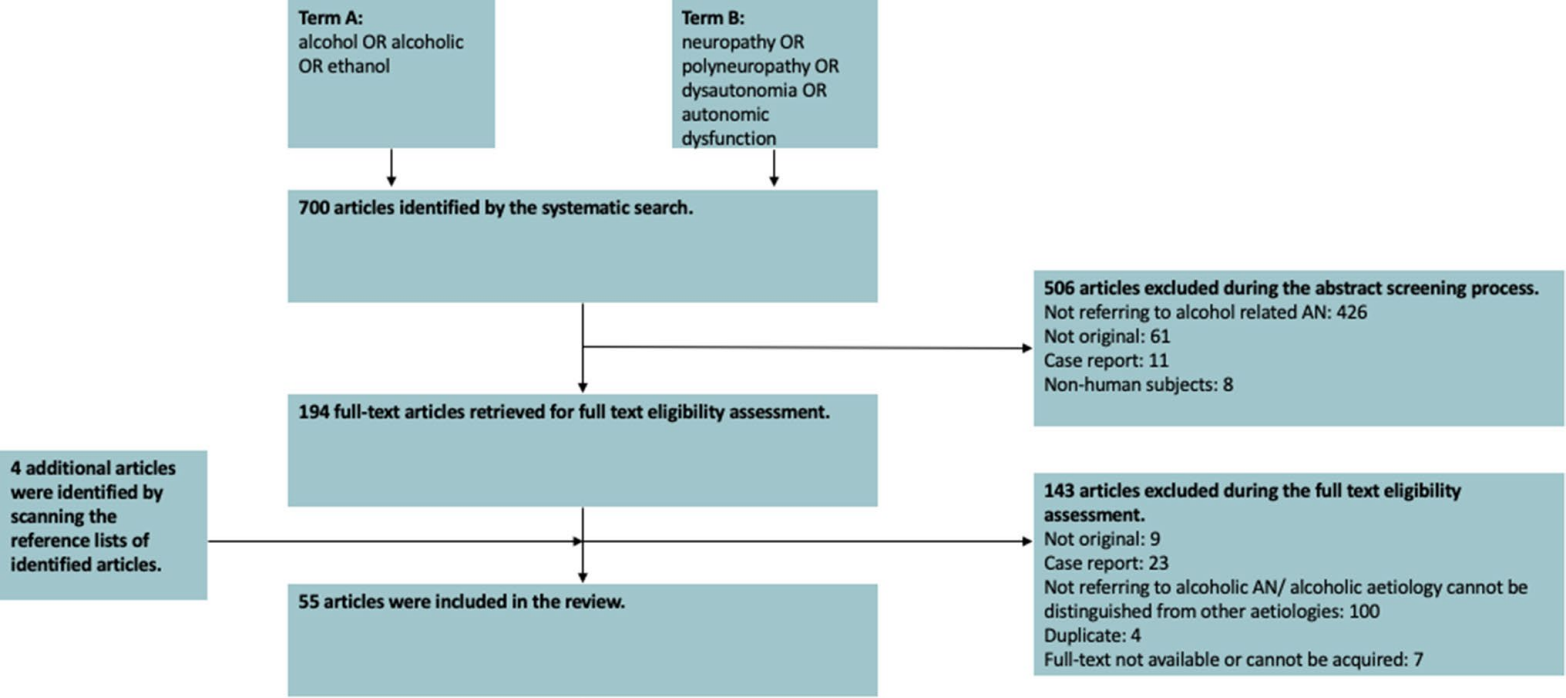
A PRISMA chart detailing the exclusion/inclusion process

The aforementioned literature search produced a total of 700 results. A total of 506 articles were excluded at the abstract screening stage. During the full paper eligibility assessment stage, a further 143 studies were excluded. Four additional studies were identified for inclusion by scanning the reference lists of the identified studies. Therefore, a total of 55 studies published between 1966 and 2018 were included in the present review. The exclusion process and reasons for exclusion are detailed in Fig. 1.

Amongst the included studies were 40 cross-sectional studies, two case-control studies, 10 cohort studies and three case series. The total population of the included studies was 2163; this total population does not include control subjects and does not include subjects who did not fit the inclusion/exclusion criteria.

### Frequency of autonomic dysfunction

#### Autonomic test batteries utilised

For the most part, the studies identified utilised non-invasive cardiovascular reflex tests in order to measure autonomic dysfunction. In general, the investigations undertaken were as described by Ewing et al. including the heart rate (HR) response to the Valsalva test, standing and deep breathing (DBT), as well as blood pressure (BP) response to standing and sustained handgrip [7]. A minority of studies measured the HR response to atropine administration, HR response to baroreceptor stimulation by external suction, and 24-h heart rate variability (HRV) [5, 8–11]. Additional non-cardiac measures of autonomic function identified in this review include sympathetic skin response (SSR) and the methacholine test for iridic innervation [12–17]. The tests identified in this review are summarised in Table 1. These investigations can be dichotomised to represent measures of sympathetic and parasympathetic function. Parasympathetic tests include HR response to DBT, Valsalva test and standing. Sympathetic tests include sweat spot test, SSR, BP response to standing and BP response to sustained hand grip. The studies providing prevalence, symptoms and the split of parasympathetic and sympathetic dysfunction are provided in Table 2. A small number of tests assessed autonomic function using laser Doppler flowmetry, which demonstrated sympathetic dysfunction relative to controls [18–20].

**Table 1:** Summary of autonomic tests utilised in papers identified within the literature

Autonomic parameter	Description
HR response to Valsalva (Valsalva ratio)	The subject blows into a mouthpiece at a pressure of 40 mmHg for 15 s. The ratio of the ratio of the longest R–R interval shortly after the manoeuvre to the shortest R–R interval during the manoeuvre is measured
HR response to deep breathing (deep breathing test)	The subject breaths at a rate of six breaths per minute. The mean of the differences between the maximum and minimum heart rate during the cycle of respiration is calculated
HR response to standing	The subject lies on a couch, then stands unaided. The ratio of the longest R–R interval around the 30th beat and the shortest around the 15th beat is calculated
HR response to atropine	IV atropine is administered. The maximum heart rate change following the infusion is calculated
HR response to baroreceptor stimulation	The carotid baroreceptors are stimulated by negative pressure applied to the subject’s neck (− 50 mmHg). The pressure is applied for a few seconds. The longest R–R interval is measured
BP response to standing	The BP is measured whilst the subject is lying down and then after standing up. The difference between these measures is calculated
BP response to sustained handgrip	Handgrip is maintained at 30% of the maximum voluntary contraction using a handgrip dynamometer up to a maximum of 5 min. The difference between the diastolic BP before release of handgrip and just before starting is taken as the measure of response
Heart rate variability	The R–R intervals are measured for 5 min at rest. The mean momentary arrhythmia is calculated
Sympathetic skin response	Electrodes are attached to the skin surface. Electrical stimuli are delivered at long, irregular intervals to avoid habituation. The voltage of the skin response is measured
Methacholine test	Pupillary diameter is measured. One drop of 2% methacholine in normal saline is instilled in each conjunctival sac. The lacrimal duct is occluded, and the pupillary diameter is remeasured after 20 min to identify constriction

The specific figures defined as abnormal are not consistent. There is some variability as to how the above tests were conducted; the table describes the most common methodology applied

**Table 2:** Studies reporting the prevalence of abnormality in autonomic function parameters

Study	Population size	Definition of alcohol abuse utilised	Autonomic parameters	Prevalence of autonomic neuropathy (%)
Rechlin et al. 1996	60	Satisfied DSM-III-R criteria for alcohol dependence	Cardiovascular reflex tests 5-min recording of HR whilst supine HR response to deep breathing, the Valsalva test and standing to calculate posture index Coefficient of variation in HR whilst resting	Cardiovascular reflex tests: 20% (≥ 3 parameters abnormal)
Weise et al. 1986	11	Fulfilled criteria of Munich alcoholism test	Mean momentary arrhythmia (HRV)	Mean momentary arrhythmia: 36% (value outside of control 95% CI)
Weise et al. 1985	31	Fulfilled criteria of Munich alcoholism test	Mean momentary arrhythmia (HRV) clinically determined by erectile dysfunction	Mean momentary arrhythmia: 16.1% (value outside of control 95% CI) Erectile dysfunction: 23% of males
Di Ciaula et al. 2016	136	Satisfied DSM-IV-TR criteria	Sweat spot test Valsalva test	Sweat spot test abnormality (sympathetic): 61% Valsalva test R–R ratio (parasympathetic): 40% (54/136)
Ferdinandis et al. 2006	23	Average daily ethanol consumption ≥ 75 g in the previous 5 years with a score of ≥ 8 in the AUDIT questionnaire	Cardiovascular reflex tests HR response to deep breathing, Valsalva test and standing BP response to standing	Cardiovascular reflex tests: 44% (≥ 2 abnormal tests) Clinical features: 9%
Nicolosi et al. 2005	40	Consumption of ethanol of 100–400 g daily for 5–25 years	Cardiovascular reflex tests HR response to DBT, Valsalva test and standing BP response to standing and sustained handgrip	‘Definite’ autonomic neuropathy: 32.5% All had both sympathetic and parasympathetic dysfunction Early ANS impairment: Additional 25% Author-defined scoring system
Ravaglia et al. 2004	132	Satisfied DSM-IV criteria	Clinical features including impotence, chronic diarrhoea, postural dizziness Cardiovascular reflex tests HR response to DBT and standing BP response to standing	Erectile dysfunction: 17% of males Other clinical signs: 0% Cardiovascular reflex tests: 26% (≥ 2/3 tests abnormal) autonomic neuropathy was parasympathetic type in 13/18 and mixed in 5/18
Agelink et al. 1998	35	Satisfied DSM-III-R criteria	Cardiovascular reflex tests 5 min resting HRV HR response to DBT and standing to calculate posture index BP response to sustained handgrip	“Possible autonomic neuropathy”: 26% (≥ 1 abnormal parameter) Parasympathetic dysfunction: 18% Sympathetic dysfunction: 23%
Monforte et al. 1995	107	At least 100 g (male) or 80 g (female) ethanol consumed a day for 2 years	Symptoms assessed including erectile dysfunction, chronic diarrhoea and postural dizziness Cardiovascular reflex tests HR response to DBT, Valsalva test and standing BP response to handgrip and standing	Postural symptoms: 11% Chronic diarrhoea: 19% Erectile dysfunction 29% of males Cardiovascular reflex tests: 24% (26/107) 18/26 of these had parasympathetic neuropathy, 8/26 had sympathetic and 3/26 were mixed
Luft et al. 1994	63	Fulfilled criteria of Munich alcoholism test	Symptoms assessed systematically Cardiovascular reflex tests HR response to standing, DBT and Valsalva test BP response to standing and handgrip	Constipation: 0% Erectile dysfunction: 2% Cardiovascular reflex parasympathetic impairment: 10% Cardiovascular reflex sympathetic impairment: 16%
Malpas et al. 1991	23	100−350 g of ethanol daily for 10–40 years	Cardiovascular reflex tests HR response to DBT, standing, Valsalva test and baroreceptor stimulation 24 h HRV	Parasympathetic dysfunction: 30% Sympathetic neuropathy: 0% (≥ 2 abnormal tests) 24 h HRV abnormality: 74%
Villalta et al. 1989	70	≥ 100 g of ethanol daily for more than 2 years	Clinical features including postural hypotension, loss of sweating ability, sphincter disturbances, erectile dysfunction, nocturnal diarrhoea Cardiovascular reflex tests BP response to standing DBT	Clinical features: 7% (5/70). 3/5 erectile dysfunction, 3/5 nocturnal diarrhoea. None had sphincter disturbance or sweating abnormality Parasympathetic (DBT): 20% Sympathetic (abnormal BP response to standing): 0%
Johnson and Robinson 1988	79	100–300 g ethanol daily for 10–40 years	Cardiovascular reflex tests BP response to standing HR response to standing Valsalva test DBT HR response to atropine	One abnormal test: 32% ≥ 2 abnormal tests: 28%
Barter and Tanner 1987	30 (note: 14 had liver disease)	≥ 80 g of ethanol daily. Mean duration of 24 years	Cardiovascular reflex tests HR response to Valsalva test, DBT and standing BP response to standing and handgrip	Cardiovascular reflex tests: 36% Parasympathetic: 16% Sympathetic: 0% Mixed: 20%
Tan et al. 1985	16	100–300 g ethanol daily for 10–40 years	Cardiovascular reflex tests HR response to standing, DBT, Valsalva test and atropine	Cardiovascular reflex tests: 19% (≥ 2 tests abnormal)
Tan et al. 1984	11	100–300 g ethanol daily for 10+ years	Cardiovascular reflex tests HR response to standing, Valsalva test, DBT and atropine	Cardiovascular reflex tests: 55%
Tan et al. 1983	10 (note: 6 had possible liver disease)	Mean of 200 g of ethanol daily for 15 years	Cardiovascular reflex tests HR response to standing, Valsalva test, DBT and atropine BP response to standing	Cardiovascular reflex tests: 40% Parasympathetic: 20% Sympathetic: 0% Mixed: 20%
Matikainen et al. 1986	28	No specific figures supplied. Recruited patients were admitted for withdrawal treatment	Cardiovascular reflex tests HR response to Valsalva test, standing and DBT BP response to standing	Parasympathetic tests Chronic alcohol abusers had reduced parasympathetic function relative to healthy controls Sympathetic tests The sympathetic nervous system was not significantly different between groups
Melgaard and Somnier 1981	14	Severe alcoholism over ≥ 10 years	The number of ‘runs’ (increasing or decreasing R–R intervals) in 150 consecutive measurements Standard deviation and mean square successive difference of the R–R intervals	No abnormalities identified compared with the control group
Duncan et al. 1980	20	100–300 g of ethanol over 12–40 years	Cardiovascular reflex tests HR response to standing, Valsalva test, DBT, baroreceptor stimulation and atropine BP response to standing	Parasympathetic neuropathy: 35% (≥ 2 abnormal tests) Sympathetic: 0%
Sebastian and Puranik 2018	30 (note: all had normal liver function tests)	Subjects had been consuming either 6 alcoholic beverages or 90 ml of ethanol a day for 5 or more years	Cardiovascular reflex tests BP response to standing, sustained handgrip and mental stress test Resting HR HR response to standing, deep breathing and Valsalva HR expiration/inspiration ratio	Parasympathetic tests Within normal range except for resting HR which was significantly increased compared to controls Sympathetic tests There was a significant increase in BP response to standing and to handgrip
Myers et al. 1979	40	No description provided	Cardiovascular reflex tests BP response to standing HR response to standing and Valsalva test Sweating abnormality in feet Methacholine test	Metacholine test: 8% Decreased sweating: 15% Cardiovascular reflex tests: 0%
Valls-Sole et al. 1990	70	≥ 100 g daily for male, ≥ 80 g for female. Must have persisted for at least 2 years	SSR	Palm SSR absent: 6% Fingertip SSR absent: 31% Foot SSR absent: 31%
Navarro et al. 1993	30	85–386 g of ethanol daily for 4–41 years	SSR SGN Cardiovascular reflex tests HR response to Valsalva test and DBT	Abnormal foot SGN: 60% Abnormal hand SGN: 23% Foot SSR absent: 53% Palm SSR absent: 13% Cardiovascular reflex tests: 73% DBT only: 40% Valsalva test only: 53%

*SSR* sympathetic skin response,* DBT* deep breathing test,* SGN* number of secreting sweat glands,* BP* blood pressure,* HR* heart rate,* HRV* heart rate variability,* ANS* autonomic nervous system

#### Cardiovascular reflex test abnormality

Amongst the identified studies, there was a significant deal of heterogeneity with respect to the total consumption of alcohol within the cohort in question, definitions of excessive alcohol intake and alcohol dependency, autonomic batteries and definitions of neuropathy utilised. Consequently, the overall prevalence of autonomic neuropathy amongst those who chronically abuse alcohol or are dependent upon alcohol cannot be produced by meta-analysis. Nonetheless, there is a significant body of research available, which supports a high rate of autonomic dysfunction amongst alcohol abusers with cardiovascular reflex measures of neuropathy producing rates ranging between 16% and 73% [12, 21]. This range is clearly quite wide and explained by the heterogeneity between studies, and it is worth noting that the most methodologically sound study with amongst the largest studied populations established that 26% of the study population (patients satisfying DSM IV criteria for alcohol dependence) have abnormal cardiovascular reflexes, defined as two or more abnormal parameters [22]. Interestingly, with respect to the cardiovascular system, parasympathetic impairment occurs with greater frequency than sympathetic, which is less common and in most studies did not occur in isolation—this is illustrated in Table 2 [8, 10, 22–26]. Currently, there are no relevant longitudinal studies with prolonged follow-up and therefore it cannot be ascertained whether this might represent a natural course in which most individuals begin with parasympathetic dysfunction and later develop sympathetic involvement as their condition progresses. Despite the significant frequency with which cardiovascular reflexes are deranged in alcohol abusers, this does not appear to translate into frequent cardiovascular features with most studies reporting patients to be asymptomatic as detailed in Table 2.

Four studies utilised Holter monitors to measure 24-h heart rate variability (HRV) to detect depressed HRV related to autonomic dysfunction. Malpas et al. found that there were subtle changes in HRV in individuals who had normal cardiovascular reflex tests, and thus proposed that HRV may indicate early ANS derangement amongst alcohol abusers [24]. Malpas et al. found a high rate of 24-h HRV abnormality, affecting 74% of subjects, 30% of which had cardiovascular reflex test abnormality. Similarly, two papers by Weise et al. used mean momentary arrhythmia to measure HRV with a lower prevalence of 16% and 36% [21, 27]. One small study by Melgaard and Somnier failed to show a significant difference in beat-to-beat variation in alcohol abusers relative to controls (*n* = 14) [28].

Presently, the extensive literature described above in relation to cardiovascular reflex tests represents the most compelling evidence that those who consume large quantities of alcohol over a prolonged period of time are vulnerable to autonomic dysfunction.

#### Sympathetic skin response abnormality

Sympathetic skin response (SSR) in chronic alcohol abusers was explored by three studies, two of which demonstrated deranged sudomotor function in alcohol abusers with absent palm SSR in 6–13%, fingertip in 31% and foot SSR in 31–53% [12, 13]. Additionally, the results support that the dysfunction occurs in the most distal segments, an observation that has been shown in patients with alcohol-related large fibre peripheral neuropathy [4]. Navarro et al. investigated the relationship between SSR and the number of pilocarpine-reactive sweat glands, finding that sympathetic involvement is associated with a decrease in their density [12]. However, conflicting these results, a study conducted by Tugnoli et al. failed to find significant differences in SSR between chronic alcohol abusers and healthy controls [14]. As the studies utilised different definitions of excessive alcohol intake, it is possible that the contrasting findings are the result of dissimilar populations. It should also be noted that of the three studies, the population size studied by Tugnoli et al. was the smallest, with 20 subjects compared with a pooled total of 100 individuals in the studies demonstrating differences between chronic alcohol abusers and healthy controls. Therefore, the evidence currently supports that deranged SSR is a feature of alcohol-related autonomic neuropathy. Clinically, however, the symptom of abnormal sweating is not one which is often highlighted as a significant feature in the literature which may indicate that this derangement is generally either subclinical or is not troubling to patients. This is illustrated in Table 2, in which most studies do not identify patients with this symptom with the exception of the article by Myers et al. in which 15% of patients had decreased sweating in the feet [17].

#### Erectile dysfunction

In males, erectile dysfunction appears to be a common feature affecting between 2–29% of chronic alcohol abusers [21, 25, 29, 30]. The relationship between alcohol abuse and erectile dysfunction was explored in greater detail by Wetterling et al. who found that there was increasing frequency of this symptom with increased alcohol consumption [31]. The wide range of the reported prevalence of erectile dysfunction in alcohol abusers was in part due to the various lengths of time between alcohol withdrawal and the commencement of the study [30]. Whilst the frequency of erectile dysfunction in this patient cohort varies significantly between studies, it is consistently the most common symptom reported.

#### Gastrointestinal features

Previously, deranged gastrointestinal (GI) physiology and a number of related clinical features which often occur in chronic alcohol abusers have been attributed to autonomic neuropathy by some authors. Though there is little literature available on the topic, what is presently available does not seem to convincingly support the role of autonomic dysfunction in such derangements. The difficulty in assessing these features arises because it is challenging to disentangle the effects alcohol exerts on gut physiology, as well as direct toxic effects to the gastrointestinal organ system and elements of derangement which are attributed to autonomic dysfunction. Alcoholism has been linked to delayed oro-cecal transit time, and gastric emptying [32–34]. Further, oesophageal dysmotility and lower oesophageal pressure changes have been identified in this population, although these findings are not consistent [35–37].

With respect to symptomatology, heavy alcohol consumption has been correlated with dyspepsia, nausea, abdominal pain and diarrhoea [33, 34]. Diarrhoea has been reported as a feature in alcohol abusers, but is not specific to the ANS [25, 29].

It is also worth noting that in studies which identified those with autonomic neuropathy using cardiovascular reflex tests, a relationship between ANS dysfunction and gastrointestinal disorder is inconsistent and consequently it currently remains unclear whether changes to the GI system in the context of alcohol abuse are related to direct toxicity to smooth muscles, nervous dysfunction or some other systemic effect [33, 34, 37].

### Risk factors

#### Total lifetime dose of ethanol

Total lifetime dose of ethanol (TLDE) is often measured in studies to establish the extent of alcohol abuse. The TLDE is calculated by taking the daily alcohol consumption multiplied by 365 and by the number of years. This measure has been explored as a risk factor for the development of autonomic neuropathy.

Of the six studies to consider TLDE a risk factor, four reported that long-term alcohol abusers with evidence of cardiovascular autonomic dysfunction had a significantly higher TLDE than those without [2, 25, 29, 38]. Two of these only identified a correlation with results from deep breathing tests [25, 29], and one found a correlation only with heart rate and blood pressure responses to standing [2]. The final paper did not identify a correlation with any specific investigation of cardiovascular autonomic dysfunctions [38]. Only one paper by Ferdinandis and De Silva was unable to find any correlation between the TLDE and cardiovascular autonomic dysfunction [39]. However, the TLDE is subject to a degree of recall bias and could be argued is particularly prone to under-reporting given the negative connotation regarding excessive alcohol consumption. Di Ciaula et al. considered gastrointestinal motility as a parameter for autonomic dysfunction and found a correlation between TLDE and gallbladder half-emptying time. They reported a lack of correlation between other measured parameters including gastric motility, dyspeptic scores and various gastrointestinal transit times [34].

#### Duration of alcohol abuse

Four studies specifically correlated the duration of alcohol abuse with presence of autonomic dysfunction through abnormal findings in cardiovascular parameters; however, the literature is conflicting [21, 22, 29, 38]. A study by Malpas et al. considered groups of chronic alcohol abusers with and without vagal neuropathies, finding no differences between these groups with respect to duration of alcohol abuse or age [24]. Furthermore, two studies showed no correlation between either cardiovascular autonomic dysfunction or abnormal sympathetic skin response and duration of alcohol abuse, although these studies did not appear to adjust for age [40, 41]. Conversely, Agelink et al. showed duration of alcoholism to be one of the most important factors contributing to the development of particularly sympathetic dysfunction, also demonstrating this independent of age [38].

#### Average daily alcohol intake

Three papers considered the effect of daily alcohol intake [29, 32, 33]. Papa et al. considered the effect of moderate and heavy alcohol consumption on intestinal transit times, finding that only heavy intake resulted in significantly increased transit times, which might indicate a visceral autonomic neuropathy. However, the authors highlighted the inability to draw a conclusion from this study as the intestinal tract is likely to also experience direct toxic effects from alcohol [32]. Showing similar results, a study considering gastrointestinal transit in chronic alcohol abusers found there was a significant correlation between mean daily consumption and gastric emptying times [33]. A single study evaluated the effects of graduating levels of alcohol consumption on cardiovascular reflex test parameters, and identified no relationship between these variables [29].

#### Hepatic dysfunction

There is debate within the literature with respect to the role of hepatic dysfunction in alcohol-related autonomic dysfunction.

Compared to healthy controls, subjects with alcoholic cirrhosis have an increased incidence of autonomic dysfunction [42, 43]. In the largest study (*n* = 107) which investigated the role of alcohol-related diseases in autonomic dysfunction, no relationship was identified between hepatic disease and autonomic dysfunction [29]. This finding is supported by a further seven smaller studies and provides strong evidence that hepatic disease is not a prerequisite for autonomic dysfunction in the context of chronic alcohol abuse [1, 22, 38, 40, 44–46]. This is further illustrated by Sebastian and Puranik who performed a study in which they compared alcohol abusers with normal liver function to healthy controls and identified significant sympathetic derangement and a higher resting heart rate but normal parasympathetic tests in those who heavily consumed alcohol, therefore further supporting the hypothesis that hepatic dysfunction is not essential to the disease process [47]. A single study of alcohol abusers with autonomic neuropathy found that these individuals are more likely to have alcoholic liver disease [26].

Five studies assessed cohorts of individuals with hepatic dysfunction of various aetiologies. In these studies, it is clear that those with liver disease have an increased prevalence of autonomic dysfunction, but that this is even higher in the context of alcoholic cause of liver disease [48–52]. This data implicates ethanol or some other confounding factor such as other health behaviours of those who abuse alcohol or a further substance contained in alcoholic beverages in the pathogenesis of autonomic dysfunction, in addition to any role which hepatic disease plays.

Weighing the available evidence, the most likely conclusion at present is that autonomic dysfunction can occur in the absence of hepatic disease and therefore the latter is not central to the disease process. However, it is feasible that hepatic disease may play a role in the autonomic dysfunction of some alcohol-abusing individuals as a co-occurring pathological factor.

#### Nutrition

Four studies commented on the role of nutrition, all showing no correlation with alcohol-related autonomic dysfunction [22, 29, 38, 53]. Three of these studies assessed nutritional status through protein and caloric intake profiles, as well as body weight [22, 29, 38]. Two of these studies excluded malnourished patients, and noted that this therefore establishes that malnutrition is not required for the presence of autonomic dysfunction [22, 38]. Monforte et al. included patients with nutritional deficiency but did not demonstrate a relationship between nutritional status and autonomic neuropathy [29]. Similarly, Koike et al. considered the role of B_12_ deficiency in other neurological disorders such as beriberi postulating that this vitamin may also impact upon the ANS in alcohol abusers, and therefore measured blood B_12_ levels to exclude B_12_ deficiency in the study population. In this study, autonomic dysfunction therefore occurred in the absence of B_12_ deficiency, excluding this as a central requirement to the pathogenesis in patients in this study [53].

At present, there is inadequate exploration of serum vitamin levels in cohorts evaluated for alcohol-related autonomic neuropathy and this prevents satisfactory assessment of this as a risk factor, or pathogenic factor for neuropathy.

#### Smoking

Only one study noted self-reported smoking habits in the context of autonomic dysfunction and found no correlation [29].

#### Age

Four studies, all assessing cardiovascular autonomic outcomes, commented on age as a risk factor [22, 24, 29, 38]. There were no significant differences between the mean ages of patients chronically abusing alcohol who did and did not demonstrate autonomic dysfunction in these studies. Three studies found no relationship; however, one study conducted by Ravaglia et al. identified a significant negative correlation with increasing age and heart rate response to deep breathing, but did not appear to have adjusted for duration of alcohol abuse or TLDE [22]. This study also considered other parameters of cardiovascular, gastrointestinal autonomic dysfunction and impotence.

#### Sex

Only two studies, both assessing only cardiovascular autonomic dysfunction, commented on sex as a risk factor. Neither found any relationship between sex and alcohol-related autonomic dysfunction [38, 40].

#### Central nervous system involvement

Interestingly, one paper commented on the increased incidence of autonomic dysfunction in patients with encephalopathy. However, as a result of the small population sizes and the conflicting results, this correlation remains uncertain [1]. Speculatively, one may consider that this risk factor could reflect a proxy for very high levels of alcohol consumption, hepatic impairment or other particularly sick state.

#### Physical fitness

Only one study commented on the relationship between physical fitness and autonomic dysfunction in alcohol abusers and found an inverse relationship [54].

#### Ethnicity

Ethnicity was not explored as a risk factor for alcohol-related autonomic neuropathy in the available literature. However, it is feasible that this may be a risk factor because of the differences between people of different ethnicities with regards to the metabolism of ethanol. Alcohol metabolism is determined primarily by the enzymes ADH, ALDH and CYP2E1 [55]. These enzymes have numerous isoforms, which are associated with different ethnicities. ADH and ALDH alleles relate to enzymes which catalyse the formation of toxic metabolite acetaldehyde from ethanol thus potentially affecting the risk of alcoholism and alcohol-related tissue damage [55, 56]. Similarly, the CYP2E1 isozyme has ethnic variance and influences the metabolism of alcohol and blood alcohol levels. However, the influence of various isozymes related to alcohol metabolism over one’s tendency to alcoholism or vulnerability to toxicity is an area of research currently considered to have results which are inconclusive [57]. Therefore, ethnicity as a risk factor may represent an interesting area for future research.

### Relationship between autonomic dysfunction and large fibre peripheral neuropathy

The literature described contrasting opinions on the relationship between autonomic dysfunction due to alcohol and alcohol-related large fibre peripheral neuropathy. A total of 35 papers commented on this relationship. The majority conclude that autonomic dysfunction and alcohol-related peripheral neuropathy can occur concurrently [2, 5, 8, 9, 11, 12, 21, 22, 25, 26, 28, 29, 33, 36, 38, 40, 49, 52, 58–60]. The largest study reporting alcohol-related large fibre peripheral neuropathy in the context of autonomic dysfunction was done by Monforte et al. which recruited 107 alcohol abusers; 41% of patients with large fibre neuropathy defined by electrophysiological criteria had autonomic dysfunction, while 15% of patients without peripheral neuropathy had autonomic dysfunction [29]. Conversely, Johnson and Robinson led a study (*n* = 79) which described 64% of patients with two or more abnormal vagal nerve function tests and 56% of those with a single abnormal vagal test results as also having peripheral neuropathy identified by clinical examination [5]. The figures often varied according to the type of autonomic dysfunction described. Gastrointestinal autonomic dysfunction did not usually occur in the presence of alcohol-related peripheral neuropathy but, as discussed earlier in the present review, autonomic dysfunction affecting the gastrointestinal system is challenging to explore [33, 36]. Only two studies found no co-occurrence between peripheral neuropathy and autonomic dysfunction [22, 44].

### Management

Presently, the only discussed management strategy is abstinence, which appears to exert a positive impact, though the evidence base is weak. Hirsch et al. studied the effect of abstinence on respiratory sinus arrhythmia, finding a significant improvement in subjects at 12 weeks follow-up (*n* = 17) [61]. Weise et al. studied the effect of abstinence on HRV in alcohol-abusing subjects with abstinence of at least 6 months, finding a significant increase in HRV after abstinence (*n* = 11) [27]. Di Ciaula et al. found that there was a significant increase in sweat spot test results after 12 months of abstinence (*n* = 136) [34]. Villalta et al. studied cardiac response to DBT following 1 year of abstinence in 12 subjects with initially abnormal results, demonstrating improvement in 11 and results reaching the normal range in nine of these [25]. Finally, Tan et al. studied cardiovascular reflex tests in chronic alcohol abusers 1–6 weeks after withdrawal, and again at 27 months, finding significant improvement in the total group and resolution of vagal neuropathy in four patients [10]. Considering these results, it can be concluded that autonomic neuropathy in the context of alcoholism may be at least partially reversible with prolonged abstinence.

### Prognosis

The data on the prognosis of alcoholic autonomic dysfunction was scarce. Several papers reported an increase in sudden death in patients with alcoholic autonomic dysfunction compared to healthy controls [5, 24, 25, 52]. However, it should be recognised that this may be just one factor in increasing relative mortality. The potential confounding effect of other alcohol-related conditions on mortality such as alcohol-related cardiomyopathy must also be considered [5, 24]. Beyond this, there is no current research which evaluates the natural history of this disease, the effect on quality of life or life expectancy.

### Assessment of bias

This review contains observational studies only. These studies are at risk of describing phenomena other than causation, and it is feasible that there is a confounder which accounts for the relationship between high alcohol consumption and autonomic dysfunction. The included studies acquired data about the total alcohol consumption of subjects through patient history, and therefore there is a high risk of social desirability or recall bias. The included study populations were frequently sourced from rehabilitation units or during admission for alcohol-related disease, and therefore there is a risk of selection bias in that this sample population may not be representative of the total population. The exclusion process in the present review was conducted independently by three authors to reduce the risk of cognitive bias on our behalf.

## Conclusions

The present review draws the following conclusions regarding alcohol-related autonomic dysfunction:

- The findings of studies included in this review with respect to the prevalence of alcohol-related autonomic dysfunction vary greatly, with some studies identifying no changes in the ANS across populations of chronic alcohol abusers and the highest prevalence identified in a population being 73%. The large range is likely indicative of the heterogeneity in the studied populations with respect to alcohol consumption or comorbidity as well as variability in the autonomic test batteries utilised by investigators.
- Cardiovascular reflex tests indicate that the parasympathetic nervous system is much more frequently affected than the sympathetic system, and that the sympathetic system is rarely affected in isolation. It is unclear whether those with parasympathetic dysfunction progress to having a mixed dysfunction with time and exposure.
- Often, autonomic dysfunction does not present with symptoms. Whilst heterogeneity in the data has prevented pooling of figures, the included studies commenting upon the symptomatic presentations of autonomic dysfunction consistently suggest that the most commonly occurring symptom in males is erectile dysfunction. Other symptoms of autonomic dysfunction are rare. At present, with a sparsity of available data it is challenging to make confident assertions as to the most common presentations of this disease.
- The most important risk factor for alcohol-related autonomic dysfunction is total lifetime dose of ethanol. This is an important observation as it demonstrates the value of alcohol cessation in the prevention of further autonomic derangement. However, there are a number of factors presently unexplored in the literature that may merit research including ethnicity, oxidative stress and type of alcohol consumed.
- There are currently few studies addressing the most appropriate management strategy for autonomic dysfunction in the context of alcohol abuse. However, there is some evidence to suggest that abstinence may lead to significant improvement.
- Alcohol-related autonomic dysfunction is positively correlated with sudden death, though it is important to note that this may be due to other confounding factors.
- Future research should aim to use more standardised measures of chronic alcohol abuse and autonomic dysfunction. Additionally, perhaps future research might benefit from elimination of subjects with comorbidities and medication use, although this is clearly challenging because of the frequency at which alcohol abusers suffer deranged liver function specifically as well as numerous other medical disorders.

## Limitations

There was an incredible deal of heterogeneity between studies included in this review with regard to the authors’ definitions of excessive alcohol consumption, choice of autonomic test batteries and definitions of autonomic dysfunction. Consequently, the data does not lend itself to meta-analysis and it is difficult to directly compare studies.

No studies identified by this review discussed the pathogenesis of alcohol-related autonomic dysfunction.

Generally, the studies identified in this review did not follow populations over time. This makes the natural course of autonomic dysfunction related to alcohol dependence difficult to elucidate.

A single database was utilised to conduct the literature search for this study. This may have caused some studies to be excluded. However, the authors checked the reference lists of every included study to identify additional seminal publications.

Of the included studies, 21/55 excluded patients taking medications which might impact upon either the autonomic nervous system, alcohol metabolism or other organ systems studied. The remaining studies did not specifically mention this criterion. Two studies acknowledged that recruited patients were indeed taking drugs which could potentially affect the reliability of the results [40, 48]. Studies of the autonomic nervous system would be most accurate in drug-naïve patients as numerous medications affect the autonomic nervous system or specific organ systems addressed in this review. Additionally, medication use may further interfere with the results of included studies because drugs can impact upon the metabolism of alcohol and therefore upon the toxic effect which ethanol consumption exerts.

Data collected by included studies such as that relating to total alcohol consumption, symptoms and medical history were generally collected by self-reported questionnaire. This data is vulnerable to error and bias as previously discussed.
